# Effects of CO_2_ and H_2_ limitations on *Methanococcus maripaludis*

**DOI:** 10.1128/spectrum.00359-25

**Published:** 2025-08-06

**Authors:** Jie Xue, Jörg Stefan Deutzmann, Nicole Matis, Frauke Kracke, Alfred Spormann, Wenyu Gu

**Affiliations:** 1 Environmental Engineering Institute IIE-ENAC, Laboratory MICROBE, Ecole Polytechnique Fédérale de Lausanne (EPFL) 27218, Lausanne, Switzerland; 2 Department of Civil and Environmental Engineering, Stanford University 6429, Stanford, California, USA; 3 Novo Nordisk Foundation CO2 Research Center, Aarhus University, Aarhus C, Denmark; 4 Department of Chemical Engineering, Stanford University 6429, Stanford, California, USA; Institute of Microbiology, Chinese Academy of Sciences, Beijing, China

**Keywords:** methanogens, starvation, reductive stress, power-to-gas

## Abstract

Hydrogenotrophic methanogens play a key role in anaerobic ecosystems by catalyzing the bioconversion of hydrogen (H_2_) and carbon dioxide (CO_2_) into methane (CH_4_) and water. This metabolic process is the basis of biological power-to-methane (PtM) technology, a promising solution for the long-term storage of surplus renewable energy as CH_4_. Its successful application can be improved through a deeper understanding of methanogen physiology, particularly the metabolic response to intermittent substrate supply of H_2_ or CO_2_. In batch cultures of PtM-relevant strains, mesophilic *Methanococcus maripaludis* and thermophilic *Methanothermobacter marburgensis*, we determined the apparent *K*_*m*_ for dissolved inorganic carbon (DIC) to be 0.60 and 1.65 mmol L^−1^, respectively, which correspond to near-maximum CH_4_ production rates *V*_max_ when CO_2_ partial pressures exceed 350 Pa at 30°C or 1.95 kPa at 60°C, at pH 7. Focusing on *M. maripaludis*, cultivation in chemostats under either H_2_ or CO_2_ limitation did not affect the yield or activity of methanogenesis. However, differences emerged when *M. maripaludis* was completely starved of either CO_2_ or H_2_. *M. maripaludis* demonstrated a higher tolerance to H_2_ starvation compared to CO_2_ starvation, resulting in shorter lag times and increased methanogenesis activity upon revival with replenished substrates. CO_2_-starved cells displayed higher intracellular F_420_H_2_/F_420_ and NADH/NAD^+^ ratios and were more susceptible to O_2_ inactivation, indicating that the accumulation of reducing equivalents may be linked to cell damage. These insights can help stabilize the operation of PtM processes by adjusting the ratio of H_2_ to CO_2_ feed during intermittent operations or shutdowns.

**IMPORTANCE:**

Microbial physiology has increasingly been studied under dynamic substrate conditions, expanding beyond the classical focus on balanced growth. While starvation is typically associated with a halt in cellular growth and activity, how cells enter starvation influences their recovery dynamics. We demonstrate that starvation caused by a lack of electron donor vs electron acceptor results in distinctly different revival behaviors in the methanogenic Archaea, *Methanococcus maripaludis*. Methanogens play a crucial role in the global carbon cycle, participating in the anaerobic breakdown of organic matter to methane and carbon dioxide. They are also of biotechnological significance, being central to anaerobic digestion processes and power-to-gas technology. Thus, our results showing improved recovery from electron donor over electron acceptor starvation may prove essential for optimizing methanogenesis processes across various applications.

## INTRODUCTION

Microbial methanogenesis is carried out by a group of strict anaerobic archaea known as methanogens (1). In particular, hydrogenotrophic methanogens reduce carbon dioxide (CO_2_) using hydrogen (H_2_), producing methane (CH_4_), water, and biomass. In this process, CO_2_ acts as the electron acceptor for cellular energy conservation and serves as the carbon source for biomass synthesis. H_2_-dependent methanogenesis is central to power-to-gas (more specifically, power-to-methane [PtM]) technology. This approach enables the storage of excess renewable electricity in the form of CH4 by utilizing H_2_ produced via water electrolysis powered by renewable energy sources. It also pertains to upgrading biogas from anaerobic waste digestion to enhance the CH_4_ concentration in the produced biogas by reducing CO_2_ (2, 3). The successful application relies on a better understanding of methanogen physiology, particularly regarding how methanogenesis activity and growth respond to varying conditions.

The US sets the natural gas pipeline grade to contain 95%–98% CH_4_ by volume (4), requiring the removal of CO_2_ and other impurities to less than 2%–5%. Ideally, methanogenesis should occur at a stoichiometric H_2_:CO_2_ ratio close to 4:1 to minimize gas impurities and ensure compliance with natural gas pipeline quality standards. However, methanogenesis in PtM depends on inherently intermittent renewable energy (5). Achieving an optimal gas composition under intermittent operation is challenging, and operational questions remain about how to handle intermittent electricity supply. One key consideration for employing methanogenesis is its CO_2_ consumption efficiency. Excess CO_2_ in the final product reduces heating value, displaces CH_4_, increases corrosion, and compromises combustion properties in biogas or synthetic natural gas (6). On the other hand, it has recently been suggested that natural gas pipeline networks can accommodate up to 5%–15% H_2_ blend as a means of enhancing the output of renewable energy systems (7, 8). However, H_2_ concentrations in the methane gas mix also need to be limited, as excessive amounts impact combustion stability and heat transfer rates and risk pipeline steel embrittlement (8, 9).

It is unclear whether and how intermittent operation would affect methanogenesis activity, particularly under CO_2_ limitation, when aiming for a CO_2_-depleted but H_2_-containing CH_4_ gas stream. In natural environments, methanogens are rarely CO_2_ limited because the CO_2_-bicarbonate system serves as the dominant pH buffer in most natural waters and is produced from organic matter degradation by respiring organisms and fermenters. Data on the CO_2_ utilization kinetics of methanogenesis are scarce. Both CO_2_ (aq) and HCO_3_^−^ are bioavailable carbon species utilized by methanogens at different stages of methanogenesis and carbon fixation (10, 11). DIC, which is the sum of [CO_2_ (aq)] and [HCO_3_–], is considered a more appropriate variable for assessing “CO_2_” limitation of methanogenesis instead of CO_2_ partial pressure (pCO_2_) (10, 12), while carbonate concentrations are negligible at neutral pH. One study reported half-saturation concentrations (*K*_*m*_) of DIC for the methanogenic strain *Methanobacterium congolense* to be 2.5 mmol L^−1^ for CO_2_ consumption and 2.2 mmol L^−1^ for CH_4_ production at pH 7 (10). The *K*_*m*_ for CO_2_ consumption in the alkalotolerant hydrogenotrophic methanogen *Methanococcus vannielii* was reported to be 0.4, 1.2, and 1.2 mmol L^−1^ DIC at pH 7, 8, and 9, respectively (12).

There has been increasing interest in understanding microbial physiology under dynamic substrate conditions. Most studies to date have focused on aerobic heterotrophs, particularly examining the impact of carbon source availability. For instance, the effects of nutrient flux (13), feast-famine cycles (14), and different carbon sources (15, 16) on cell death rates and revival dynamics have been investigated. Limited studies investigated the effect of substrate intermittency in biological methanation (17) and the PtM process (18, 19), where both H_2_ and CO_2_ supplies are intermittent (17), or where the electrons supplied from electrochemical systems are intermittent (18, 19), or in mixed cultures (17, 18). However, the influence of different substrates, electron donors, and acceptors has not been studied or compared.

In this work, we aim to investigate the effects of H_2_ vs CO_2_ limitation and starvation in the model hydrogenotrophic methanogen *Methanococcus maripaludis*, also as a PtM-relevant strain owing to its high specific methane production rate in laboratory-scale *in situ* PtM systems (19–23). To define the threshold for CO_2_ limitation and starvation, we determined the apparent half-saturation constants of CO_2_ (*K*_CO2_) utilization through batch cultures. We additionally reported *K*_CO2_ for the thermophilic *M. marburgenesis* as a commonly used strain in pilot-scale *ex situ* PtM systems (2, 24), which exhibited a comparable affinity for low CO_2_ concentrations. Next, focusing on *M. maripaludis*, we analyzed how CO_2_ vs H_2_ limitation influenced the performance of methanogenesis in chemostats under steady-state conditions. Lastly, we explored the effects of CO_2_ vs H_2_ complete starvation on the survival and revival of *M. maripaludis*, revealing that the cells experienced greater stress during CO_2_ starvation. Based on F_420_H_2_/F_420_ and NADH/NAD^+^ measurements, as well as oxygen (O_2_) stress experiments, we conclude that this physiology is linked to a more reduced intracellular state.

## RESULTS

### Uptake kinetics of CO_2_ for methanogenesis

To understand the circumstances under which methanogenic activity could be limited by DIC, we determined DIC uptake kinetics for two model methanogens in closed-batch cultures using serum bottles. Defined amounts of sodium bicarbonate were added to CO_2_-depleted cultures of *M. maripaludis* and *M. marburgenesis* cultures under conditions of excess H_2_. Notably, the cultures were starved for less than 12 hours before the bicarbonate addition experiments, which caused minimal effects on subsequent methanogenesis performance (see below). Using the time-series data of DIC concentration decrease and methane formation, apparent affinity constants (*K_m_*) for DIC were determined by applying ordinary differential equations (ODEs). The apparent *K_m_* for DIC of *M. maripaludis* and *Methanothermobacter marburgensis* was determined to be 0.60 (95% confidence interval [CI]: 0.48–3.83) mmol L^−1^ and 1.65 (95% CI: 1.44–2.92) mmol L^−1^ (Fig. S1 and S2).

### Methanogenesis by *M. maripaludis* under H_2_- or CO_2_-limited growth in chemostats

To investigate whether CO_2_ limitation would impact the methanogenesis rate or biomass yield compared to the more commonly observed H_2_ limitation, *M. maripaludis* was cultivated in chemostat reactors using either H_2_ or CO_2_ as the limiting substrate (see Supplemental figures). Data were collected from at least four independent replicate bioreactors for each condition during steady-state balanced growth. Biomass yields were 1.41 ± 0.22 g DW mol^−1^ CH_4_ vs 1.49 ± 0.27 g DW mol^−1^ CH_4_ for H_2_ and CO_2_ as the limiting substrates, respectively, while specific methanogenesis rates were 40.8 ± 10.2 mmol CH_4_ h^−1^ g DW^−1^ and 44.1 ± 15.6 mmol CH_4_ h^−1^ g DW^−1^, respectively (Fig. S3), indicating no significant difference in biomass yield and methanogenesis rate between the two conditions.

### Starvation and reactivation of *M. maripaludis* under H_2_- and CO_2_-depleted conditions

**Fig 1:**
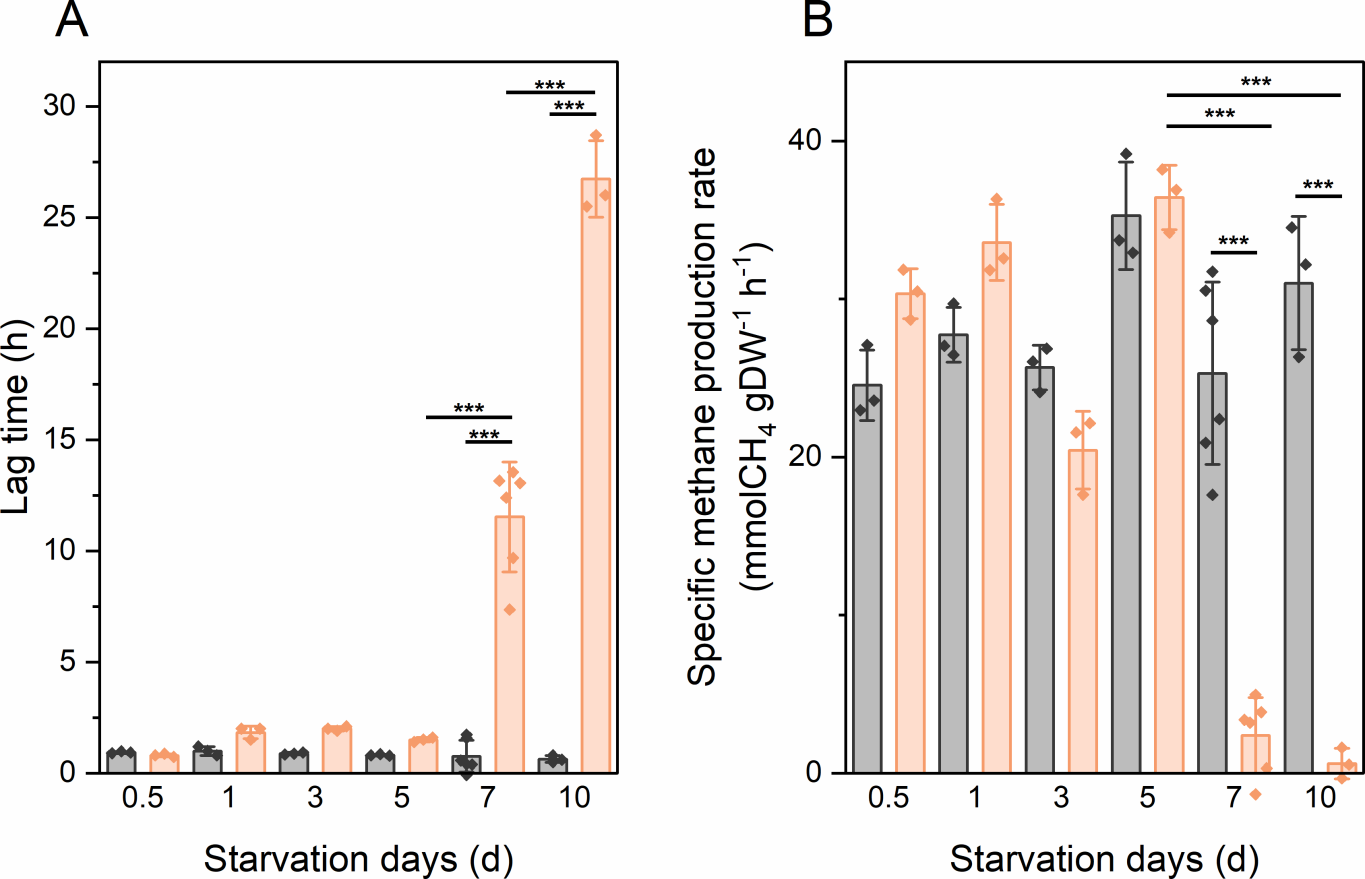
Revival kinetics as lag time (**A**) and specific CH_4_ production rate within initial 5 hours of revival (**B**) of *M. maripaludis* cells after H_2_ starvation in the presence of 10 kPa CO_2_ (condition a, gray bars) or CO_2_ starvation in the presence of 81 kPa H_2_ (condition b, orange bars) from 0.5 to 10 days. Error bars represent the SD of data from three to six biological replicates. Significance tests were performed to compare H_2_ and CO_2_ starvation and between starvation days for each condition. Stars indicate a significant difference. *: *P* < 0.05, **: *P* < 0.01, and ***: *P* < 0.001, based on one-way analysis of variance (ANOVA) followed by *post hoc* analysis using Tukey’s multiple comparisons test. Data for 7- and 10-day starvation under condition b were significantly different from all other conditions in both panels A and B but are not shown for clarity.

To investigate the metabolic response to intermittent substrate availability, we compared the effects of extreme H_2_ and CO_2_ limitation, i.e., starvation, on *M. maripaludis*. After halting the chemostats, we observed that cells subjected to CO_2_ limitation and subsequent CO_2_ starvation exhibited longer lag times upon restart. To further examine this, we tested the survival of *M. maripaludis* during prolonged H_2_ vs CO_2_ starvation and subsequent reactivation in closed-batch serum bottle cultures under (i) H_2_-starved cultures with excess CO_2_ (pCO_2_ = 10 kPa) (condition a) and (ii) CO_2_-starved cultures with excess H_2_ (pH_2_ = 81 kPa) (condition b). To explore the reactivation dynamics, H_2_ and CO_2_ were resupplied after subjecting *M. maripaludis* to different periods of H_2_ or CO_2_ starvation. Significantly longer lag phases and lower initial specific CH_4_ production rates were observed after 7 days of CO_2_ starvation compared to H_2_-starved cells (Fig. 1). This effect is less pronounced after starvation for fewer than 5 days, as cells maintained the ability to resume growth and methane production at high rates with a short lag period. In contrast, H_2_-starved cells are readily active and revivable within an hour, even after 10 days of starvation.

**Fig 2:**
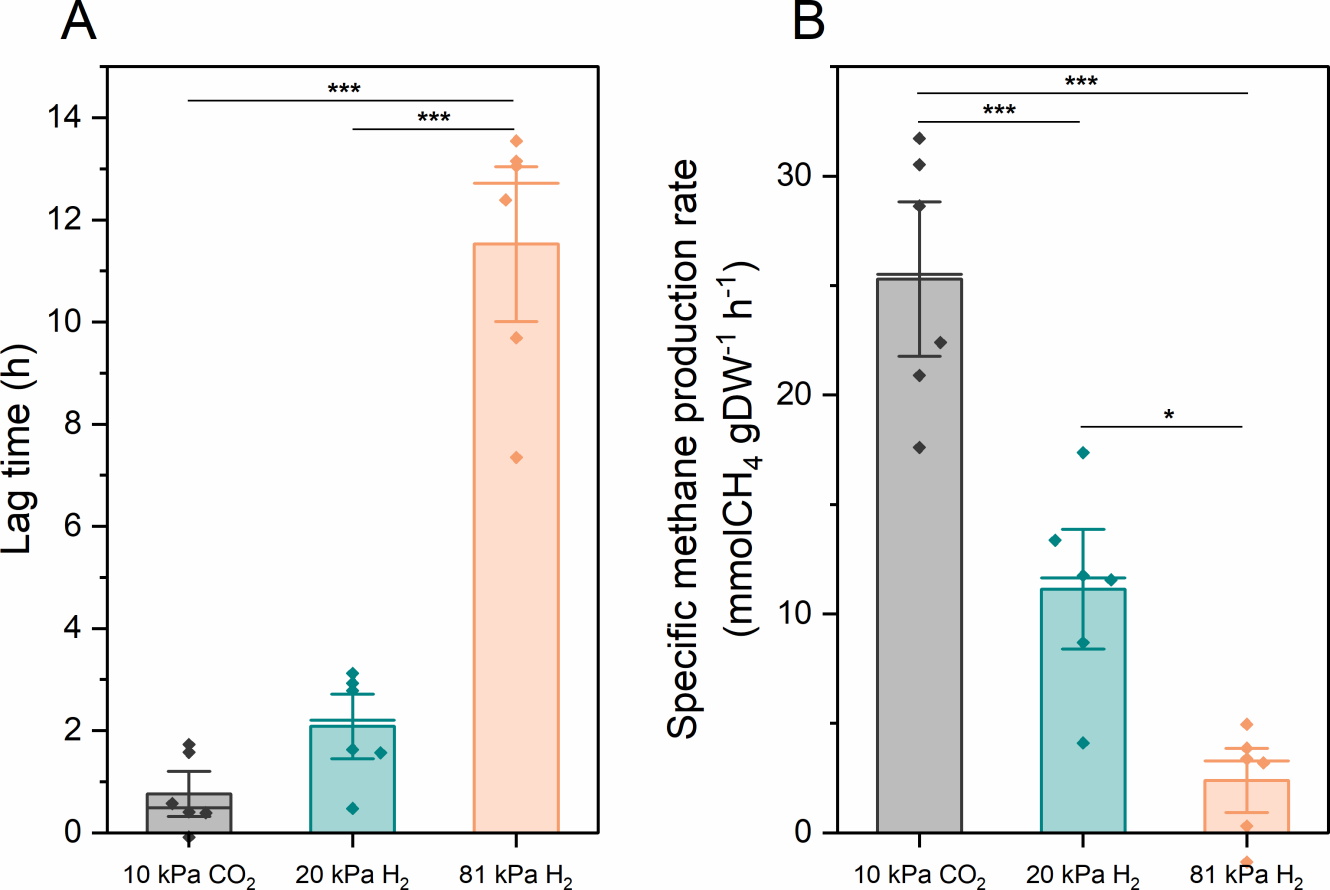
Lag time (**A**) and specific CH_4_ production (**B**) of *M. maripaludis* during the revival phase after 7 days of H_2_ or CO_2_ starvation. Black: condition a, H_2_ starvation in the presence of 10 kPa CO_2_; green: condition c, CO_2_ starvation in the presence of 20 kPa H_2_; orange: condition b, CO_2_ starvation in the presence of 81 kPa H_2_. Representative data from six biological replicates are plotted. One-way ANOVA followed by *post hoc* analysis using Tukey’s multiple comparisons test was performed. Stars indicate significant differences. *: *P* < 0.05, **: *P* < 0.01, and ***: *P* < 0.001.

To investigate whether the presence of H_2_ during CO_2_ starvation causes an inhibitory effect in a concentration-dependent manner, we introduced a third condition, c: CO_2_-starved cultures with lower H_2_ partial pressure (pH_2_ = 20 kPa). A starvation period of 7 days was selected, during which the lag times between conditions a and b were significantly different in the previous experiment. We hypothesized that a lower pH_2_ would alleviate this difference. Indeed, the effect of H_2_ on recovery from CO_2_ starvation was less pronounced at lower concentrations (Fig. 2). After 7 days of starvation, cells under condition a exhibited a lag time of 0.76 ± 0.72 hours, cells under condition b exhibited a lag time of 11.53 ± 2.47 hours, while cells under condition c displayed a shorter lag time of 2.08 ± 1.03 hours (Fig. 2A). The lag time for condition c is significantly shorter than for condition b. A similar trend was observed for the specific CH_4_ production rates after ending the starvation conditions (Fig. 2B). Therefore, cells not only revived more slowly but were also less active during the initial revival phase from CO_2_ starvation, particularly when the H_2_ partial pressure was high during starvation.

**Fig 3:**
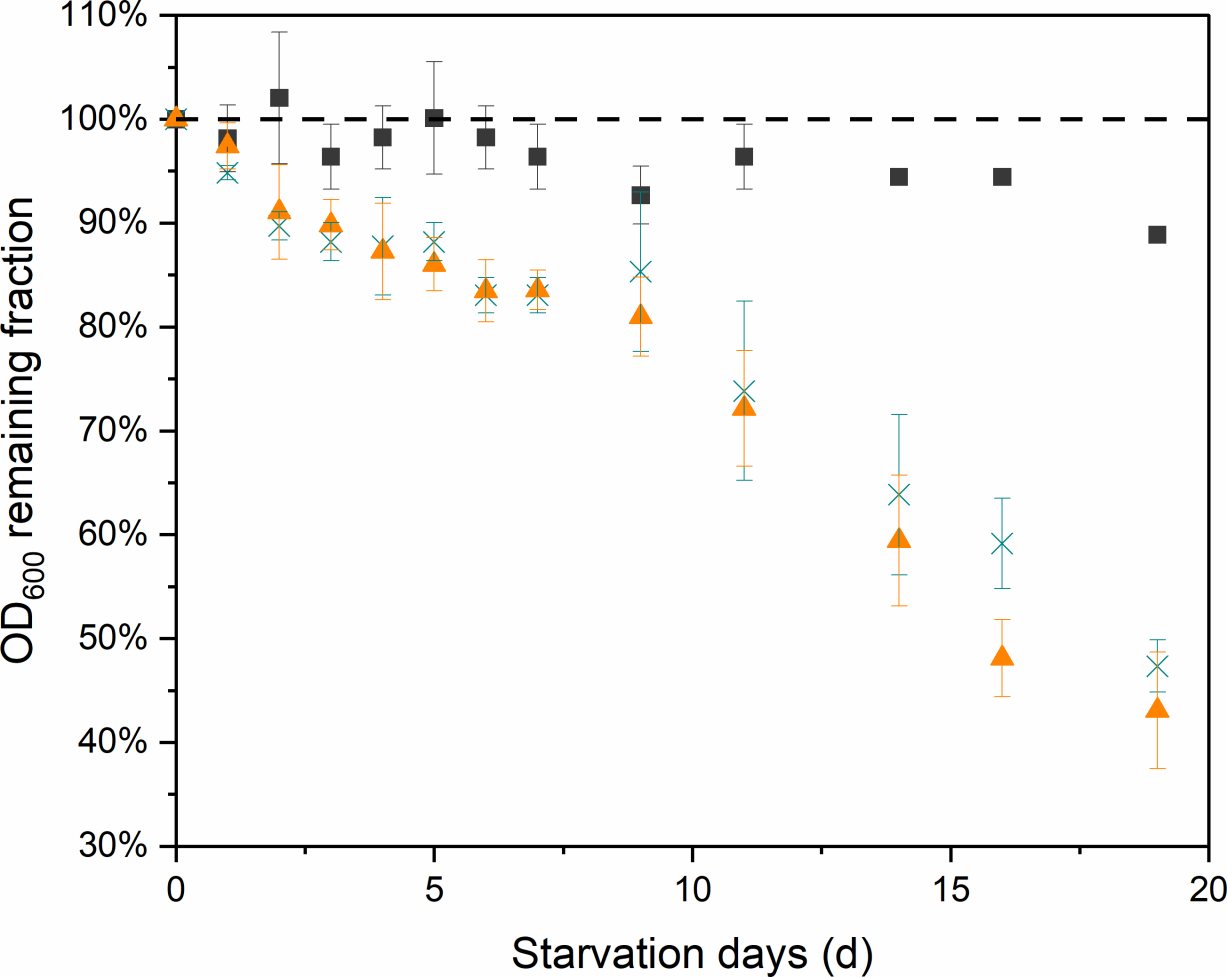
OD_600_ decrease of *M. maripaludis* cells during H_2_ or CO_2_ starvation for 20 days. Dashed line represents no cell lysis and maintenance of 100% of the initial OD_600_. Black squares: condition a, H_2_ starvation in the presence of 10 kPa CO_2_; orange triangle: condition b, CO_2_ starvation in the presence of 81 kPa H_2_; green cross: condition c, CO_2_ starvation in the presence of 20 kPa H_2_. Error bars represent the SD of data from at least three biological replicates.

As *M. maripaludis* cells lyse rapidly after death (21, 25), we also monitored the OD_600_ decrease in *M. maripaludis* cultures over a 20-day starvation period under conditions a, b, and c, where the starved cultures were incubated shaking at 30°C. OD_600_ decreased from its initial value under all three conditions (Fig. 3). After 19 days, H_2_-starved cultures exhibited a low decrease of OD_600_ (condition a, 11.1% ± 3.2%), whereas the OD_600_ of CO_2_-starved cells decreased by more than 50% (condition b: 56.9% ± 0.06%; condition c: 52.6% ± 0.03%; Fig. 3). The OD_600_ decrease in conditions b and c was significantly higher than under condition a (statistical significance student’s *t*-test *P* = 0.0046, *P* = 0.0007, respectively). Notably, there is no significant difference between conditions b and c, indicating that lowering the H_2_ partial pressure did not alleviate the effect of cell lysis in the presence of H_2_.

### High pH_2_ leads to cells in a more reduced state and inhibits metabolic reactivation

**Fig 4:**
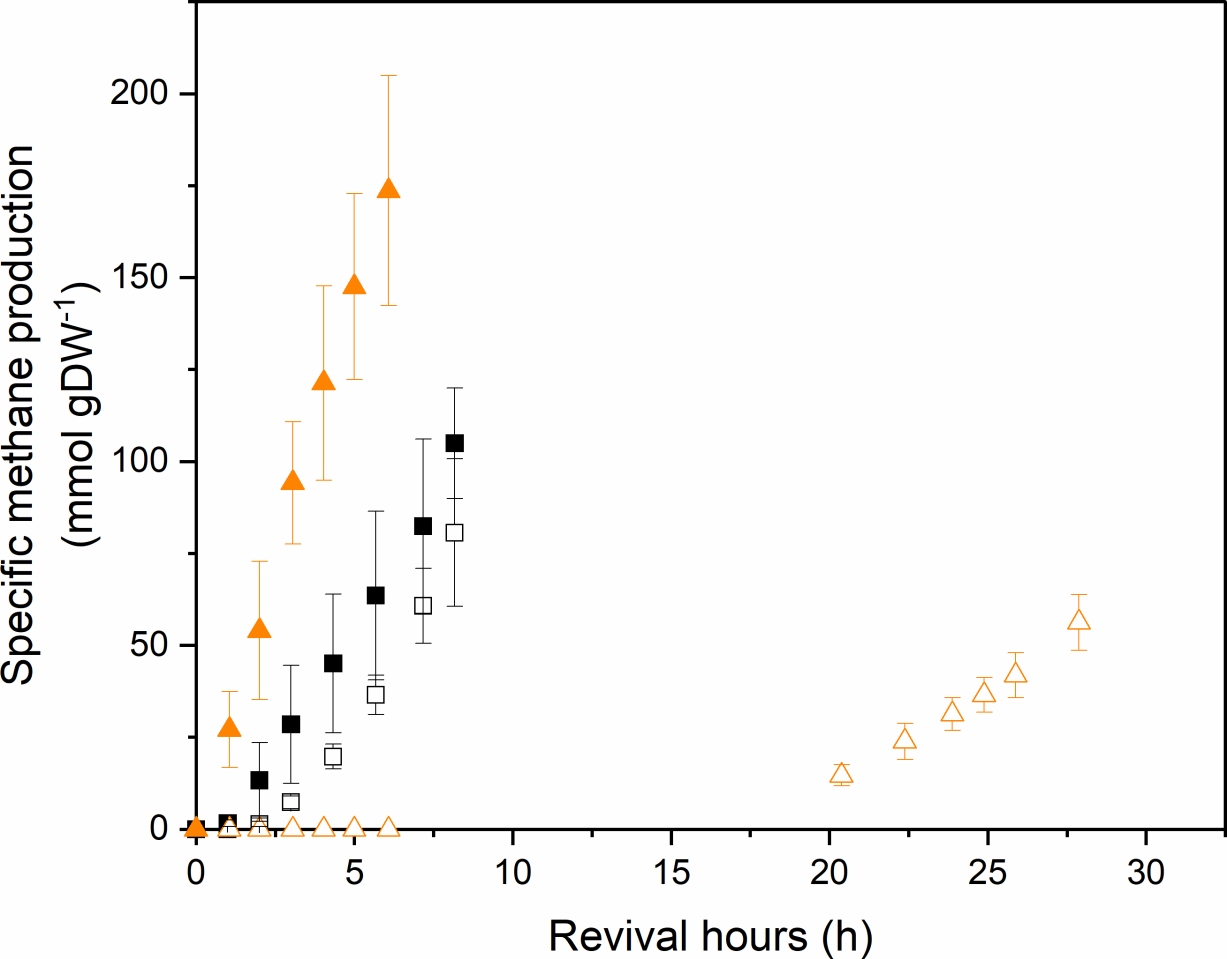
Specific CH_4_ production of *M. maripaludis* during the revival phase after 3 days of starvation with or without O_2_ exposure. Black squares: condition a, H_2_ starvation in the presence of 10 kPa CO_2_; orange triangles: condition b, CO_2_ starvation in the presence of 81 kPa H_2_. Open symbols: O_2_ exposure for 1 hour; filled symbols: no O_2_ exposure. Error bars represent the SD of data from at least three biological replicates.

To test whether high H_2_ concentration during CO_2_ starvation results in “more reduced cells,” we exposed the starved cells to O_2_ for 1 hour and monitored their reactivation. More reduced cells may produce a greater amount of reactive oxygen species (ROS) when exposed to O_2_. We selected a starvation period of 3 days, during which the lag times between conditions a and b were not significantly different (Fig. 2). We hypothesized that O_2_ exposure would preferentially hinder the reactivation of CO_2_-starved cells, leading to differences in lag times. After 3 days of starvation in the presence of either 10 kPa CO_2_ or 81 kPa H_2_ (conditions a and b), the control samples without O_2_ exposure exhibited a lag time of less than 1 hour, with comparable CH_4_ production rates under both conditions, which are consistent with previous observations (Fig. 1 and 4). Following O_2_ exposure, a marked inhibition of CH_4_ production was observed during the first 4 hours of revival of cells that had been starved in the presence of 10 kPa CO_2_ (condition a). However, the rates quickly recovered to levels similar to the control after 5 hours (Fig. 4). In contrast, the cells starved in the presence of 81 kPa H_2_ (condition b) and exposed to O_2_ experienced a lag time of over 15 hours, and the subsequent CH_4_ production rate was significantly slower (Fig. 4). Cells exposed to O_2_ showed no increase in OD_600_ after 25 hours (Fig. S4), likely due to severe cellular damage caused by ROS.

**Fig 5:**
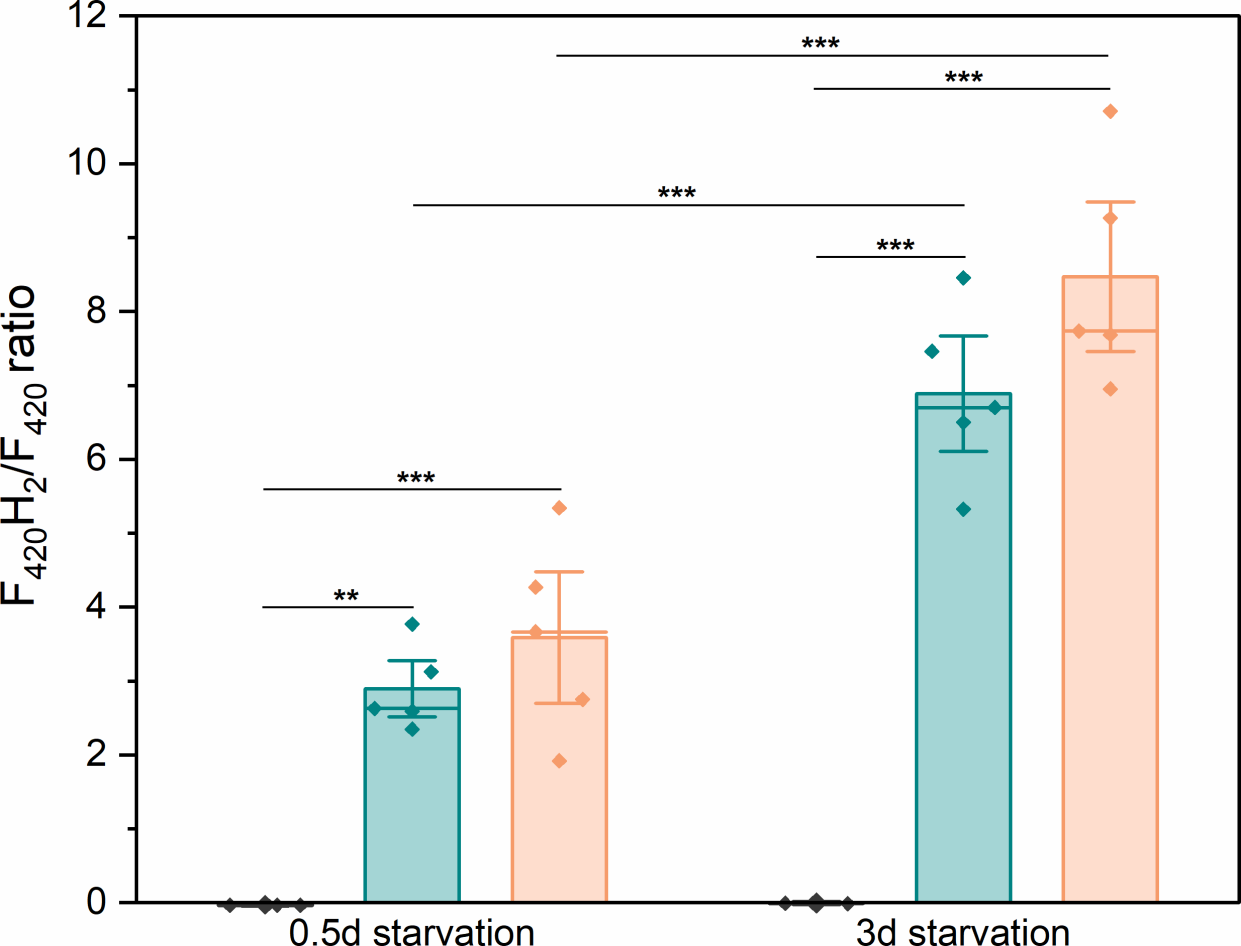
F_420_H_2_/F_420_ ratios in *M. maripaludis* on 0.5 and 3 days after the onset of starvation, under three gas conditions. Gray: condition a, H_2_ starvation in the presence of 10 kPa CO_2_; green: condition c, CO_2_ starvation in the presence of 20 kPa H_2_; orange: condition b, CO_2_ starvation in the presence of 81 kPa H_2_. Error bars represent the SD of data taken from five biological replicates. Tukey’s multiple comparisons test was performed to compare H_2_ and CO_2_ starvations and starvation days for each condition. Stars indicate significant differences. *: *P* < 0.05, **: *P* < 0.01, and ***: *P* < 0.001.

Subsequently, we sought to directly measure the intracellular F_420_H_2_/F_420_ and NADH/NAD^+^ ratios in cells under conditions a, b, and c as indicators of the intracellular redox state both at the onset of starvation (within 12 hours) and after 3 days of starvation. During exponential growth, the F_420_H_2_/F_420_ ratios under all three gas conditions remained similar, with an average of 3.1 ± 1.4. After entering starvation, at both 0.5 and 3 days of starvation time, the F_420_H_2_/F_420_ ratios showed distinct patterns depending on headspace gas composition. Under 10 kPa CO_2_ (a), the ratios at both 0.5 days and 3 days remained close to zero, significantly lower than those under H_2_-containing conditions, indicating that nearly all F_420_ were oxidized (Fig. 5). In contrast, under 81 kPa H_2_ (b), the ratio increased significantly from 3.6 ± 1.3 at 0.5 days to 8.5 ± 1.5 at 3 days (Fig. 5). Similarly, under 20 kPa H_2_ (c), the ratio rose from 2.9 ± 0.6 to 6.9 ± 1.2 (Fig. 5). These results suggest that in the presence of H_2_, F_420_ predominantly exists in the reduced form and progressively accumulates during prolonged starvation.

In addition to F_420_H_2_/F_420_, intracellular levels of NAD+ and NADH were also quantified. Although NADH is a minor electron carrier in methanogen cells, the ratio of its redox couples still indicates cellular state and serves as a complementary check to the F_420_H_2_/F_420_. Commercial kits are available for absolute quantification (details see Supplemental figures). A similar trend was observed for the NADH/NAD^+^ ratio. At both 0.5 and 3 days, the ratio under condition a was significantly lower than those under conditions b and c. Moreover, the NADH/NAD^+^ ratios under H_2_-containing conditions b and c increased significantly after three days, gradually shifting toward a more reduced intracellular redox state (Fig. S5).

## DISCUSSION

The apparent affinity constant for DIC reported here for *M. maripaludis* and *M. marburgensis* is 0.60 (95% CI: 0.48–3.83) mmol L^−1^ and 1.65 (95% CI: 1.44–2.92) mmol L^−1^, respectively, indicating their capacity to reduce CO_2_ at 50% of their maximum rate when pCO_2_ exceeds ca. 350 Pa at 30°C and 1.95 kPa at 60°C, assuming bicarbonate buffer system equilibrium and at pH 7.0. *M. maripaludis* possesses a putative bicarbonate ABC transporter encoded by loci MMP0529, which is an active transporter requiring ATP and could aid DIC uptake at low concentrations (26). Both strains also harbor putative carbonic anhydrase encoded by loci MMP1299 in *M. maripaludis* and MTBMA_c01680 in *M. marburgensis* (NCBI), which catalyze the bidirectional conversion of CO_2_ and H_2_O into HCO_3_^−^ and protons H^+^, thus facilitating DIC conversion to CO_2_ as a substrate for methanogenesis (11). DIC as a metric for electron acceptor limitation is deemed appropriate (10, 12).

These *K*_CO2_ values are similar to the reported *K_m_* in *M. congolense* (10) and *M. vannielii* (12), confirming affinities for DIC in the low millimolar range, in contrast to the micromolar range of affinity for H_2_ (27, 28). The comparatively high *K_m_* for CO_2_ aligns with the generally high CO_2_ concentrations found in methanogenic environments, where CO_2_ levels in anaerobic digesters typically range from 25 to 51 kPa (29). Therefore, inorganic carbon availability would not restrict the methanogenic activity of *M. maripaludis* under standard operating conditions, for instance, in biogas production, where other factors, like pH, may exert a more significant influence on methanogenic rates (12). When upgrading biogas to >98% CH_4_ (<2% CO_2_), these *K*_CO2_ values suggest that the methanogenesis rates would be limited to 50%–88% and 37%–54% of *V*_max−CH4_ for *M. maripaludis* and *M. marburgensis*, respectively, at their corresponding growth temperatures and neutral pH (calculated according to the equation: *V*/*V*_max_ = *S*/[*K_m_* + *S*] based on the CI of *K*_*m*_).

Previous studies of methanogenesis in chemostats were mostly conducted under electron donor limitations of either H_2_ (30) or formate (25). In cases of anabolic substrate limitation by phosphate or leucine (30, 31), a decoupling of biomass synthesis from methanogenesis was observed, where a higher ratio of carbon from CO_2_ is converted to CH_4_ rather than biomass. Our results suggest that *M. maripaludis* exhibits similar catabolic and anabolic activity under steady-state conditions during electron acceptor limitation as it does during electron donor limitation (Fig. S3). However, the cells are more resistant to H_2_ starvation than CO_2_ starvation under dynamic substrate conditions. This could have implications for process designs that rely on the intermittent availability of renewable power and CO_2_ sources. Exposure of resting cells to H_2_ resulted in cell lysis and a decline in cellular activity during the initial revival phase, suggesting compromised cellular or enzymatic integrity (Fig. 1 to 3). The prolonged lag phase seems to be caused by a combination of fewer intact cells and less activity of the remaining cells. This effect also appears dose-dependent based on H_2_ partial pressure (Fig. 2 and 3). This observation may highlight an intrinsic characteristic of methanogens, as they likely face only H_2_ limitation in their environmental niches (32). H_2_, generated as an intermediate product during the degradation of organic matter by some fermenting microbes, contrasts with CO_2_, which is produced by most respiration and fermentation processes, as well as from the atmosphere, and is unlikely to be limiting in nature.

Evidence from O_2_ exposure tests, F_420_H_2_/F_420_, and NADH/NAD^+^ ratios measurements indicates that environmental H₂ exposure increases the ratio of reduced species within the cells, affecting the cellular redox balance likely mediated by hydrogenases. During O_2_ exposure of CO_2_-limited cells, we hypothesize that the more reduced state inadvertently promotes the formation of ROS, leading to damage of cellular components such as FeS-containing enzymes and DNA, which may explain the lower cellular activity during the revival phase. It is also important to consider the effects of medium components, especially the metals and reduced compounds (cysteine) present, as they could promote the formation of ROS, thereby affecting cell viability (33). However, since the medium composition was the same for H_2_- and CO_2_-starved cells during O_2_ exposure, we attribute the difference mainly to the differing redox states of the cells. Methanogens utilize several redox cofactors, including ferredoxins (34), coenzyme F_420_ (35), and NAD(P)H (36), for central metabolism and biosynthesis. F_420_ is a key electron carrier in the methanogenesis pathway, and the F_420_H_2_/F_420_ ratio is a major indicator of the intracellular redox state (1, 37). Our measured F_420_H_2_/F_420_ ratios during the exponential growth phase are similar under all three gas conditions (*P* > 0.5) and are consistent with previously reported ratios of 2–5 in exponentially growing cells under 4:1 H_2_/CO_2_ headspace (35). This indicates that *M. maripaludis* actively maintains an intracellular redox balance when both electron donors and acceptors are sufficient. Once the electron donor H_2_ was close to depletion (condition a), the redox balance shifted toward the oxidized state. Upon entering starvation, the excess electron acceptor CO_2_ and lack of H_2_ quickly led to nearly complete oxidation of F_420_ (Fig. 5). In contrast, the depletion of the electron acceptor CO_2_ did not immediately cause a major redox shift on the onset of starvation as compared to during the exponential phase. As starvation progressed, the redox balance gradually shifted toward a more reduced state, accumulating reduced F_420_H_2_ and a corresponding increase in the F_420_H_2_/F_420_ ratio. We propose that the redox imbalance, characterized by a shift toward a more reduced state, may affect the intracellular physiological environment, impairing survival during starvation and cellular activity during a recovery phase.

The effect of substrate starvation likely results from a combination of changes in intracellular redox balance and severe energy limitation. Previous studies have investigated gene expression changes during the balanced growth of *M. maripaludis* cells in H_2_-limited chemostats. Compared to phosphate or nitrogen limitations, H_2_ limitation leads to increased expression of genes involved in the methanogenic pathway utilizing F_420_, including F_420_-reducing hydrogenase (Fru), F_420_-dependent methylenetetrahydromethanopterin dehydrogenase (Mtd), and F_420_-dependent methylenetetrahydromethanopterin reductase (Mer) (38, 39). This suggests that cellular redox functions are regulated by H_2_ availability. Motility has also been correlated with electron donor limitation and growth rate, likely linked to H_2_ chemotactic responses in *M. maripaludis* (25, 40). No stress response or regulatory mechanisms have been described under these conditions (38–40), and *M. maripaludis* generally maintains a relatively static proteome (25, 31). The mechanism of H_2_-induced cell lysis and activity impairment during complete starvation remains unclear and warrants further investigation.

In applications such as PtM, methanogenesis may operate with intermittent H_2_ and/or CO_2_ supplies, exposing cells to intermittent fasting. Our findings provide evidence from a cellular physiology perspective that *M. maripaludis* cell integrity is better preserved under H_2_ limitation compared to CO₂ limitation. However, maintaining low H_2_ pressure during periods of intermittency can help support cellular integrity and facilitate a more rapid resumption of activity upon re-supplementation of substrates. It is interesting to investigate whether this observation also holds true for other hydrogenotrophic strains, such as the thermophilic *M. marburgensis*, as a commonly selected species in PtM. This insight is also beneficial for laboratory practices; adding a small amount of bicarbonate to the media can help ensure that cells remain H₂-limited after growth, thereby supporting their viability. These findings expand our understanding of how substrate dynamics influence microbial survival and revival, with electron donor vs acceptor limitation differentially impacting intracellular redox states.

## MATERIALS AND METHODS

### Microbial strains and cultivation

The methanogen strain *M. maripaludis* MM901 (41) was cultured in high salt minimal medium, either “JD” modified for an electrochemical system with reduced chlorine concentration (20) or a simplified McN medium (42). Both media were prepared anoxically and adjusted to pH 7.0 ± 0.2. Cultures were maintained under H_2_:CO_2_ (4:1 [vol/vol]) gas mix at 30°C with 200 rpm shaking. The JD medium contains Na_2_SO_4_ 210 mmol L^−1^, MgSO_4_ 16.2 mmol L^−1^, KH_2_PO_4_ 0.8 mmol L^−1^, NH_4_Cl 4.5 mmol L^−1^, CaCl_2_ 0.3 mmol L^−1^, Na-resazurin solution 0.1% (wt/vol), SL10 trace element solution (43), selenite-tungstate solution (44), and 1 mL per liter, buffered by 50 mmol L^−1^ morpholinepropanesulfonic acid (MOPS). The simplified McN medium contains KCl 2.3 mmol L^−1^, MgSO_4_ 16.2 mmol L^−1^, NH_4_Cl 4.5 mmol L^−1^, CaCl_2_ 0.5 mmol L^−1^, NaCl 330 mmol L^−1^, K_2_HPO_4_ 0.8 mmol L^−1^, selenite-tungstate solution (44), SL10 trace element solution (43), Na-resazurin solution 0.1% (wt/vol), and Wolin’s vitamin solution (DSMZ medium 141), buffered by 100 mmol L^−1^ MOPS. Reducing reagents, L-cysteine HCl 2 mmol L^−1^, thiosulfate 4 mmol L^−1^, and sodium sulfide 2 mmol L^−1^, were added after autoclaving.

The methanogen strain *M. marburgensis* Marburg (DSM 2133, DSMZ, Germany) was maintained in a low salt minimal medium under the same gas environment at 60°C and 200 rpm shaking. The medium was adjusted to pH 7.0 ± 0.2, containing 30 mmol L^−1^ K_2_HPO_4_, 20 mmol L^−1^ NaH_2_PO_4_, 40 mmol L^−1^ NH_4_Cl, and 10 mL trace element solution (0.47 mmol L^−1^ nitrilotriacetic acid, 0.84 mmol L^−1^ MgCl, 0.16 mmol L^−1^ FeCl_2_, 0.003 mmol L^−1^ CoCl_2_, 0.019 mmol L^−1^ NiCl_2_, and 0.002 mmol L^−1^ NaMoO_4_), additionally buffered with 50 mmol L^−1^ MOPS. The medium was reduced by 1.2 mmol L^−1^ Na_2_S.

Both strains were maintained in sealed glass serum bottles, regularly supplied with an H_2_:CO_2_ gas mixture, and transferred for maintenance every few weeks.

### Analytical methods

The gaseous compounds CO_2_, CH_4_, and H_2_ were quantified using a gas chromatograph (8610C, Agilent, USA, or MG#5, SRI Instruments, USA) as described previously (20). Cell densities (OD_600_) were measured using a CO8000 cell density meter (WPA, or Ultrospec 10110, Biochrom, UK). A coefficient of 0.34 g DW OD^−1^ L^−1^ was used to convert OD_600_ to dry weight (25).

### Experimental setup for determining *K*_CO2_ for DIC

To determine the *K*_*m*_ for DIC, serum bottles containing 25 mL of JD medium for *M. maripaludis* or low salt medium for *M. marburgenesis* were prepared without CO_2_ or bicarbonate. The 135 mL headspace was filled with 100% H_2_ to ambient atmospheric pressure. The bottles were then inoculated with 2.5 mL of mid-exponential phase preculture, and a 4:1 H_2_:CO_2_ gas mixture was added to 69 kPa overpressure (on top of the H_2_). In this way, H_2_ remains in excess during the experiments. The methanogen cultures were incubated shaking for 20 hours overnight to deplete CO_2_, while H_2_ was in excess, which was confirmed by gas chromatography (GC). The OD_600_ of cultures at this stage ranged between 0.21 and 0.47 for both strains. At this stage, and during the following experiments, the cultures did not reach any other nutrient limitation besides CO_2_, as indicated by higher biomass resulting from CO_2_ re-addition.

Subsequently, the headspace was flushed with 100% H_2_ to remove the produced CH_4_ and adjust the headspace pressure to ambient atmospheric pressure. The CO_2_-depleted cultures received various amounts of sodium bicarbonate from a 150 mmol L^−1^ stock solution (maintained in a 100% CO_2_ headspace). A wide range of bicarbonate additions (0.06–0.81 mmol) was used to ensure the robustness of the measurements and subsequent model fitting, leading to a total of 14 and 27 groups of data sets for *M. maripaludis* and *M. marburgensis*, respectively (Fig. S1). The cultures were immediately monitored by GC for gas phase CO_2_ and CH_4_ at 15–20 minute intervals until CO_2_ was no longer detectable in the gas phase (detection limit is approximately 50 ppm, in equilibrium with DIC = 90 nmol L^−1^ at pH 7.0 and 30°C), lasting 2–6 hours. During these experiments, the medium was buffered with MOPS as described above, and the bicarbonate addition resulted in a negligible pH change. In these experiments, cell densities were not closely monitored, as preliminary tests revealed that frequent liquid sampling introduced technical variability in gas measurements in the low concentration range in the CO_2_ depletion phase.

The measured gas phase CO_2_ concentrations were then converted to DIC concentrations in liquid using Henry’s constants for CO_2_ of 30.4 mmol L^−1^ atm^−1^ (saltwater at 30°C) and 14.8 mmol L^−1^ atm^−1^ (water at 60°C) (45), with the first dissociation constant being pKa_1_ = 6.33 (46). The calculation and subsequent fitting rate parameters involve several assumptions and approximations: (i) [CO_2_ (g)] is in equilibrium with [CO_2_ (aq)] and [HCO_3_^–^ (aq)] at a given time, under constant temperature and neutral pH, while [CO_3_^2–^(aq)] is neglected. (ii) CH_4_ has low solubility in the aqueous phase, and soluble CH_4_ is neglected. (iii) The gas phases of the batch cultures maintained >90% H_2_ at ambient atmospheric pressure during the experiment, and the bottles were constantly shaken; thus, H_2_ was assumed not to be limiting.

### Fitting rate parameters

Both DIC consumption and CH_4_ production data were used to fit Monod kinetic parameters (47, 48). The system is described by the following equations:


(1) $$\mu =\frac{\mu_{\text{max}}\;S}{K_{s}+S}$$

(2) $$\frac{dX}{dt}=\mu_{\text{max}}X$$

(3) $$X=X_{0}+\beta Z$$

(4) $$Z=\alpha (S_{0}-S)+Z_{0}.$$

Equation 1 is the Monod kinetics, where μ_max_ is the maximum specific growth rate, *K*_*s*_ is the half-saturation constant for growth. Equation 2 defines the biomass growth rate, where *X* denotes the biomass concentration. Equation 3 describes that biomass production and CH_4_ production (*Z*) are proportional, with β as the proportionality constant. Equation 4 indicates that CH_4_ production and DIC consumption are also proportional, with α as the proportionality constant. Subscript “0” indicates the initial values of the respective parameters. After simplification:


(5) $$\frac{dS}{dt}=-\frac{\mu }{\alpha }\left(Z+\frac{X_{0}}{\beta }\right).$$

Equations 1, 4, and 5 were used for ODE fitting, involving only observed variables *S* and *Z*. The global parameters μ_max_, *K*_*m*_, and α were fitted across all data sets, while *Z*_0_, *S*_0_, and “*X*_0_/β” were fitted per experimental group.

To estimate the parameters numerically, the coefficient α was first estimated through linear regression of DIC decrease vs CH_4_ production across all data sets for each strain. We obtained the ratio of –Δ mole CH_4_/Δ mole (CO_2_ [g] + CO_2_ [aq] + HCO_3_^−^ [aq]) as 0.85 (95% CI: 0.82–0.88) for *M. maripaludis*, and 0.86 (95% CI: 0.84–0.89) for *M. marburgensis*. These agree with reported values that generally 90% of substrates are converted to CH_4_ in methanogenesis (25, 31).

Using α, the remaining parameters were fitted via ODE simulation (scipy.integrate.odeint) and estimated by minimizing a weighted least squares loss using “scipy.optimize.least_squares.” Residuals between model predictions and observed *S* and *Z* were calculated. To ensure that the fitting treats groups consistently and that the larger observed values do not dominate the loss function, we introduce normalization of the residuals by scaling by the maximum observed values for both *S* and *Z* for each group. A weighting of 50% was applied equally to *S* and *Z* residuals. In this way, our fitting does not distinguish *K*_CO2_ for methanogenesis activity (CH_4_ production) and CO_2_ uptake as previously done (49). Additionally, a regularization penalty term is added to the loss function to minimize the variance of group-specific X_0_/β parameters, driven by the fact that the starting biomass (OD_600_) is close between groups, reducing overfitting within individual groups. To assess the model performance, the *R*^2^ of both *S* and *Z* overall is reported.

To assess parameter uncertainties, bootstrap resampling was conducted by resampling experimental groups in proportion to their number of data points (with replacement), maintaining the original total number of data points. For each bootstrap sample, model fitting was reapplied, and the fitted parameter values were gathered. The 95% CI was calculated based on bootstrap percentiles. μ_max_ was estimated at 0.16 (95% CI: 0.08, 0.49) h^−1^ for *M. maripaludis*, consistent with previous characterizations of the range of 0.2–0.5 h^−1^ (25, 50). For *M. marburgensis*, μ_max_ was estimated at 1.05 (95% CI: 0.78, 1.45) h^−1^, with the lower bound of the 95% CI exceeding the previously reported value of 0.69 h^−1^ (51).

### Starvation of *M. maripaludis* by CO_2_ and H_2_ and revival

The starvation-revival dynamics of *M. maripaludis* were studied in serum bottles, batch cultures using the modified McN medium with 3 mmol L^−1^ L-cysteine HCl as the reducing agent. Each condition was performed with 3–6 biological replicates. To minimize the interference of gas change, the headspace gas composition was pre-set according to different starvation conditions before inoculation. For H_2_-starvation (condition a), the headspace composed of atmospheric level of 101 kPa N_2_ and 50 mmol L^−1^ NaHCO_3_ was added to the medium as CO_2_ supply. For CO_2_-starvation, the headspace was composed of either 101 kPa H_2_ (condition b) or a mixture of 68 kPa N_2_ and 33 kPa H_2_ (condition c), resulting in starvation conditions under 81 kPa H_2_ and 20 kPa H_2_, respectively, without the addition of NaHCO_3_.

Cells from an active *M. maripaludis* culture were inoculated into serum bottles. A 4:1 H_2_:CO_2_ gas mixture was added to 69 kPa on top of the preset atmospheric level gas phases. A few such additions allowed the cultures to reach OD_600_ of 0.2–0.4 before initiating starvation tests. The concentrations of H_2_ and CO_2_ in the headspace were constantly monitored, and the onset of starvation was defined as the time point when either gas fell below the GC detection limit.

After starvation periods of 0.5, 1, 3, 5, 7, and 10 days, revival was initiated by providing 69 kPa of a 4:1 H_2_:CO_2_ gas mixture. CH_4_ concentration was monitored every half hour for approximately 5 hours to calculate the initial methanogenesis rate, and OD_600_ was observed for up to 20 hours to assess lag phases. The duration of the lag phase was determined by fitting two linear segments to the log scale of the OD_600_ data: one for the lag phase with no detectable growth and another for the exponential growth phase. The intersection of these two fitted lines marked the time point indicating the end of the lag phase.

### O_2_ exposure experiments

Active *M. maripaludis* cultures under starvation conditions a and b, prepared as described above, were starved for 3 days. Subsequently, the headspace was replaced with air at ambient pressure through flushing, and the cultures were shaken at 30°C for 1 hour. Next, the cultures were flushed with N_2_ for 30 minutes and supplemented with 3 mmol L^−1^ L-cysteine HCl to restore anoxic conditions and provide a reduced sulfur nutrient source. The headspace was filled with 69 kPa of a 4:1 H_2_:CO_2_ gas mixture to initiate revival. Headspace CH_4_ concentration and OD_600_ were monitored until the exponential phase was observed.

### Coenzyme F_420_ measurement

The reduced to oxidized F_420_ ratios were determined following the established procedures with minor modifications (37). Cells were cultured under the three conditions described above and harvested for F_420_ analysis at the onset of starvation (<0.5 days) and after 3 days. At least five biological replicates were performed for each condition.

Cultures in serum bottles were placed into an anaerobic glovebox. A 1 mL sample was extracted with syringes, mixed with 0.5 mL of anoxic acetone, and immediately pipetted into a 96-well plate for fluorescence detection of F_420_H_2_. Anoxic acetone was prepared by sparging with N_2_ and left overnight in the glovebox. Fluorescence was measured using a BioTek Synergy H1 Multimode Reader (Agilent, US) at room temperature with 427 nm excitation and 471 nm emission. For each sample, three technical replicates of 200 µL were loaded into separate wells for detection. This allowed the measurement of oxidized F_420_, which exhibits blue fluorescence when excited at 420 nm. The reduced form, F_420_H_2_, is non-fluorescent and was quantified indirectly by oxidizing F_420_H_2_ to F_420_. To determine the total F_420_ concentration, a separate aliquot of each sample was exposed to air for 3 minutes with vigorous mixing. Subsequently, 0.5 mL of aerobic acetone was added, mixed, and the fluorescence was measured. The ratio of reduced to oxidized form F_420_H_2_/F_420_ was calculated using the formula (total F_420_ – oxidized F_420_)/oxidized F_420_. The fluorescence intensity was background-corrected by subtracting the fluorescence of a 2:1 mixture of blank medium and acetone. The excitation spectra, ranging from 340 nm to 450 nm, were recorded and inspected to assess measurement quality. Additionally, we observed a rapid decrease in fluorescence signal over time, potentially due to the photosensitivity or degradation of F_420_. Therefore, fluorescence was measured immediately after mixing the sample with acetone to minimize signal loss.

## SUPPLEMENTAL MATERIAL

Supplemental figures spectrum.00359-25-s0001.docx Fig. S1 to S6.

ASM does not own the copyrights to Supplemental Material that may be linked to, or accessed through, an article. The authors have granted ASM a non-exclusive, world-wide license to publish the Supplemental Material files. Please contact the corresponding author directly for reuse.
