# Supplementary material for: Effects of CO2 and H2 limitations on Methanococcus maripaludis
Source: Microbiol Spectr. 2025 Aug 6;13(9):e00359-25. doi: 10.1128/spectrum.00359-25 (PMC12403587; doi:10.1128/spectrum.00359-25)
Supplement: Supplemental figures — Fig. S1 to S6. [file spectrum.00359-25-s0001.docx]

Supplementary information for manuscript:

Effects of CO_2_ and H_2_ Limitations on *Methanococcus maripaludis*

Jie Xue,^a^ Jörg Stefan Deutzmann,^b,c^ Nicole Matis,^b,c^* Frauke Kracke,^b†^ Alfred Spormann,^b,c,d^ Wenyu Gu^a#^

^a^Environmental Engineering Institute IIE-ENAC, Laboratory MICROBE, Ecole Polytechnique Fédérale de Lausanne (EPFL), Lausanne, Switzerland

^b^Department of Civil and Environmental Engineering, Stanford University, Stanford, California, USA

^c^Novo Nordisk Foundation CO_2_ Research Center, Aarhus University, 8000 Aarhus C, Denmark

^d^Department of Chemical Engineering, Stanford University, Stanford, California, USA

Running Head: Methanogenesis under CO_2_ or H_2_ limitations

^#^ Address correspondence to Wenyu Gu, [wenyu.gu@epfl.ch](mailto:wenyu.gu@epfl.ch).

^†^ Current address: Science at Stripe Climate and Frontier, 354 Oyster Point Blvd, South San Francisco, USA

Keywords: methanogen, starvation, reductive stress, Power-to-gas

Operation and monitoring of *M. maripaludis* chemostat reactors

Parallel continuous flow chemostats were operated using modified McN medium, with either H_2_ or CO_2_ as the limiting substrate. H_2_-limited reactors received a 3:1 H_2_:CO_2_ gas mix along with 50 mM sodium bicarbonate in the medium. CO_2_-limited reactors were supplied with 100% H_2_ gas and 30 mM sodium bicarbonate. Additionally, 20 mmol L^-1^ of NaCl was added to maintain consistent salinity between the two conditions.

The reactor vessels comprised 150 ml Pyrex medium bottles fitted with gas-tight rubber stoppers. Each reactor was filled with 50 ml of medium under anoxic conditions and magnetically stirred. The reactors were continuously sparged with gas, regulated by flowmeters (VWR, US). A reservoir medium bottle containing anoxic medium was connected via a peristaltic pump, supplying medium to the reactor bottle at a constant rate of 0.11 ± 0.01 h^-1^ for H_2_-limited reactors and 0.13 ± 0.03 h^-1^ for CO_2_-limited reactors. The effluent liquid was collected in waste bottles for monitoring the flow rate. Volumetric gas flow rates of the reactor off-gas were measured using a Milligas counter (Ritter Apparatebau, Germany). All reactors were operated at ambient pressure and 30 °C. OD, pH, liquid and gas flow rates, and concentrations of H_2_, CO_2_, and CH_4_ were monitored daily. Reactors were considered to be in steady state after stable operation for four retention times.

To ensure H_2_ limitation, we increased the mass transfer of the 3:1 H_2_:CO_2_ gas mixture by raising the stirring speed and observed an increase in biomass in the reactors. For CO_2_ limitation, we ensured that the CO_2_ concentration in the gas phase was below the detection limit of the gas chromatograph. Additionally, increasing the stirring speed in the presence of 100% H_2_ gas did not result in an increase in biomass, indicating the absence of H_2_ limitation. However, adding more sodium bicarbonate to the liquid medium increased the biomass content, proving that no other nutrient was limited, but only CO_2_.

NAD^+^, NADH measurements

Sample preparation and the measurement of NAD⁺ and NADH concentrations were performed using a commercially available kit according to the manufacturer’s recommendations (NAD/NADH-Glo™ Assays, Promega, US) (1). Triplicate samples were collected from cultures under three different starvation conditions, both at the onset of starvation and after three days of starvation. The preparation of headspace gases, as well as the growth and starvation phases, adhered to the same procedures described in the starvation-revival dynamics experiments.

For each sample, 40 mL of culture was centrifuged at 4°C at 12,000 g. The cell pellet was washed with 3 mL of phosphate-buffered saline. The cells were then resuspended in 50 μL of phosphate-buffered saline, followed by the addition of 50 μL of 0.2 mol L^-1^ NaOH solution containing 1% dodecyltrimethylammonium bromide to lyse the cells. Two aliquots of 50 μL from the lysed cell samples were transferred to a 96-well plate; one 50 μL aliquot was mixed with 25 μL of 0.4 mol L^-1^ HCl. The 96-well plate was then heated at 60 °C for 15 minutes to degrade either NADH or NAD⁺ in one of the two 50 μL lysed cell samples. After cooling to room temperature for 10 minutes, the acid-treated samples were neutralized by adding 25 μL of 0.5 mol L^-1^ Trizma base solution, and 50 μL of a solution containing 0.2 mol L^-1^ HCl and 0.25 mol L^-1^ Trizma base was added to the acid-free samples. Next, 100 μL of the NAD/NADH-Glo™ Detection Reagent was added to each well, gently mixed, and incubated at room temperature for 45 minutes. Luminescence data were collected using a microplate reader (BioTek, Agilent, US), and the concentrations of NAD⁺ and NADH were calculated from the standard curve prepared in the same plate according to the manufacturer’s instructions. The calculated concentrations were converted to the concentration in the cultures and normalized by cell dry weight. Depending on the homogeneity of variance among the data groups, either Tukey’s multiple comparisons test or a two-sample t-test with Welch's correction was applied.


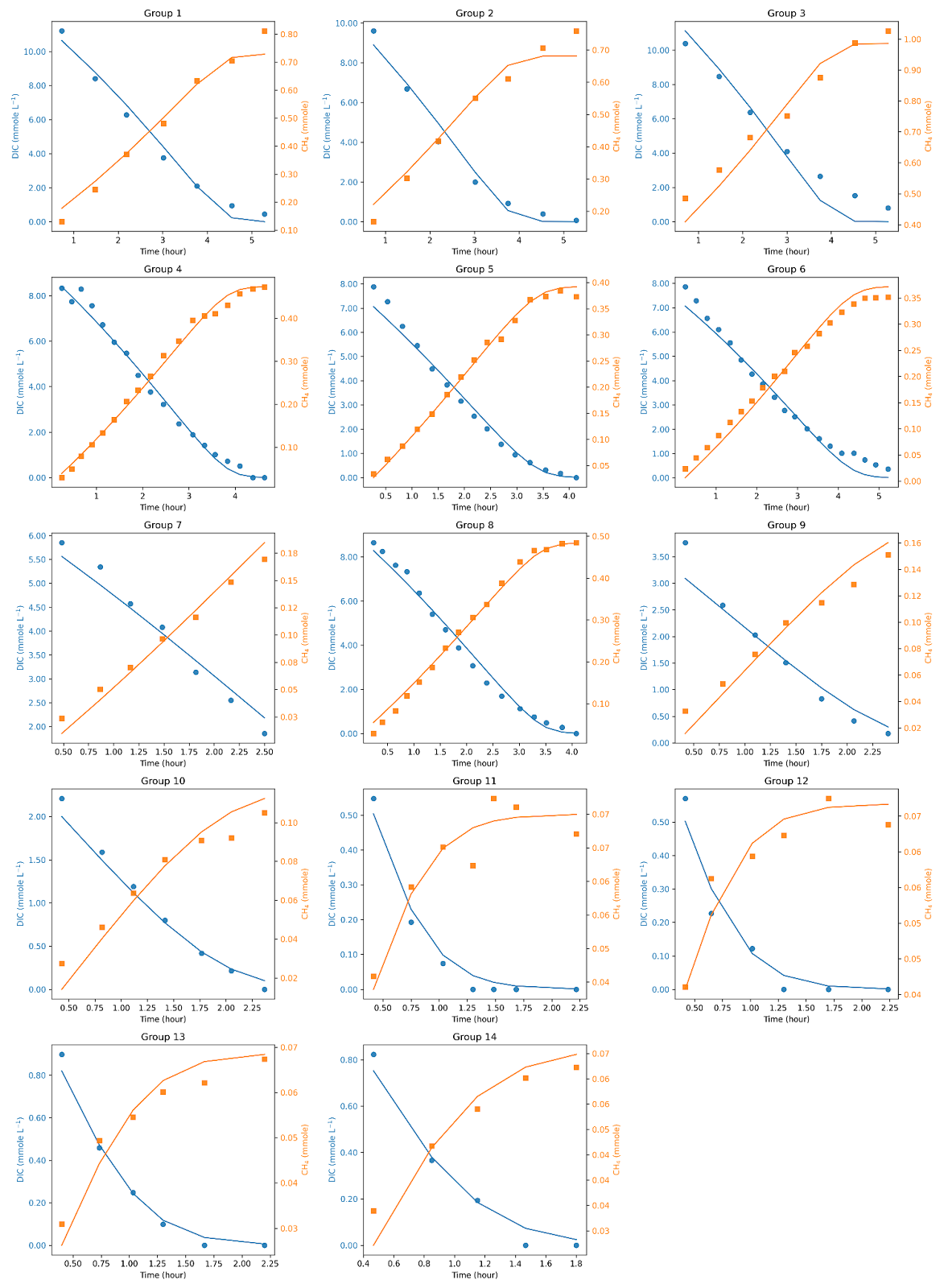


Figure S1. Time course of DIC consumption (blue circles) and CH_4_ production (orange circles) in *M. maripaludis* cultures for K_s_ determination. Blue and orange lines represent the model-fitted curves for DIC and CH_4_, respectively. The overall R² for DIC consumption fitting is 0.98 and for CH_4_ production fitting is 0.99.


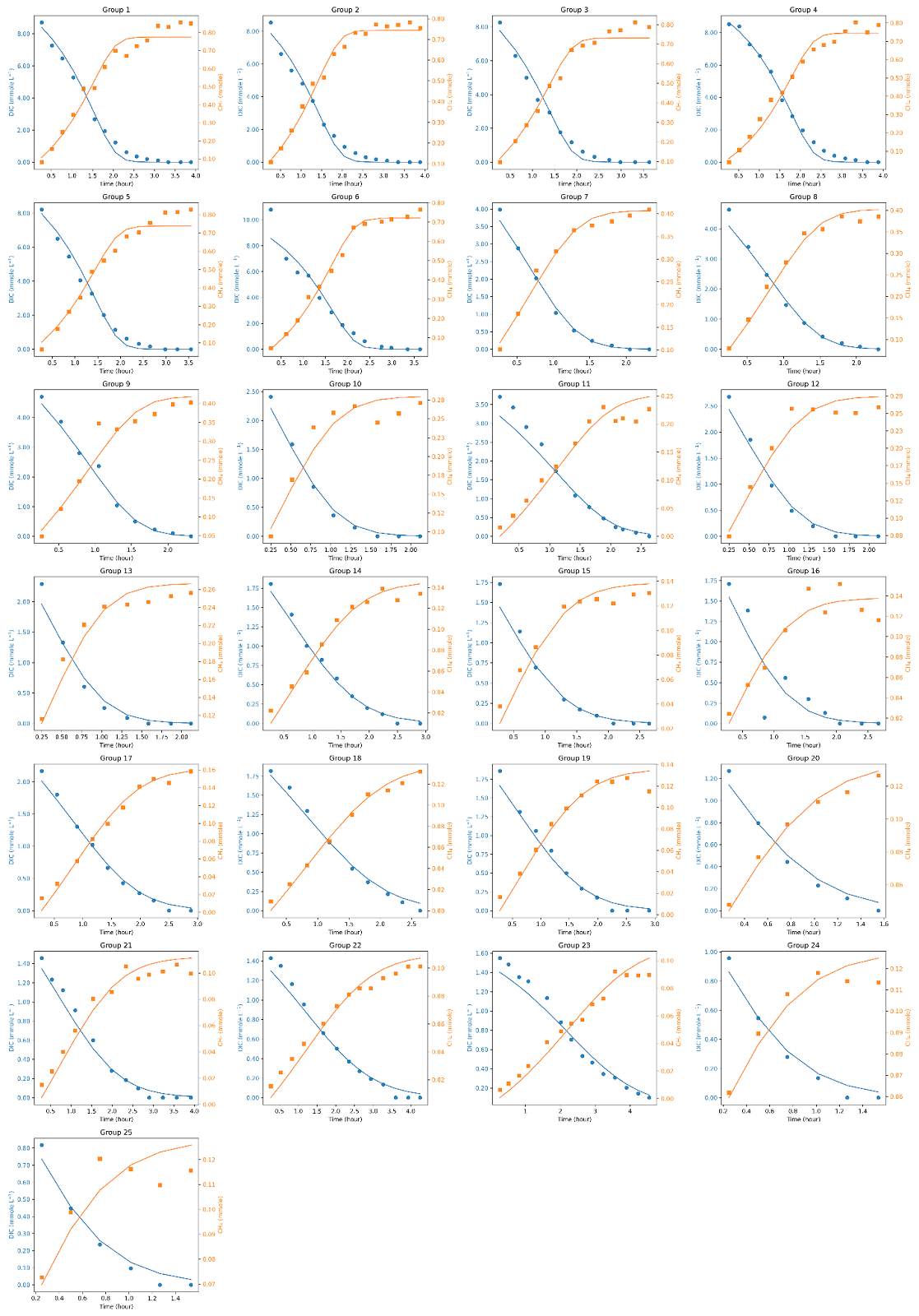


Figure S2. Time course of DIC consumption (blue circles) and CH_4_ production (orange circles) in *M. marburgensis* cultures for K_s_ determination. Blue and orange lines represent the model-fitted curves for DIC and CH_4_, respectively. The overall R² for DIC consumption fitting is 0.98 and for CH_4_ production fitting is 0.99.


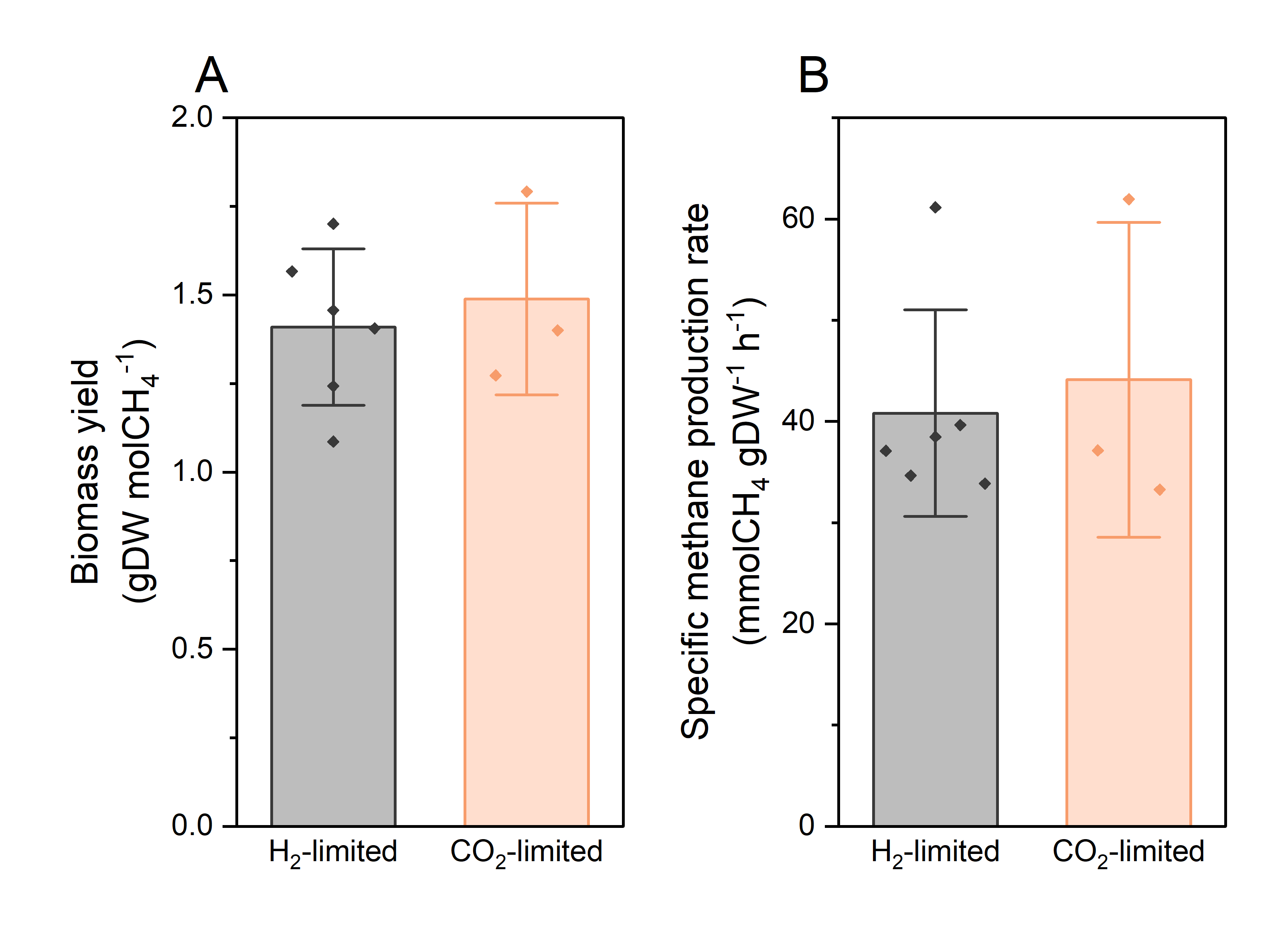


Figure S3. Biomass yield (A) and specific methane production rates (B) of *M. maripaludis* in chemostat under H_2_ (grey bars) *vs.* CO_2_ (orange bars) limitations. Error bars represent the standard deviation of data from at least three replicate chemostat reactors. Individual data points are shown as diamonds.


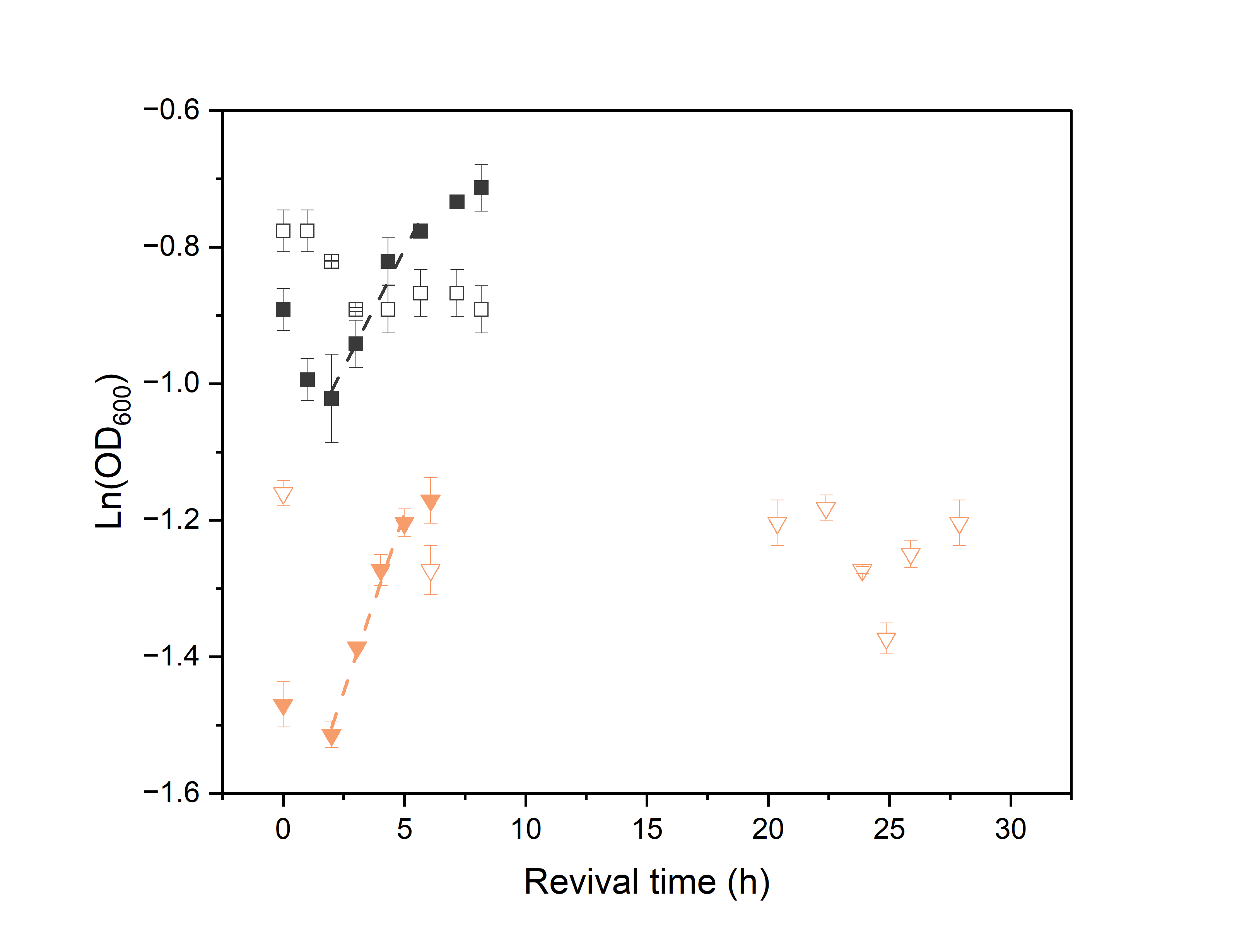


Figure S4. Ln(OD_600_) of *M. maripaludis* during the revival phase after 3 days of starvation with or without oxygen exposure. Black squares: condition a, H_2_ starvation in the presence of 10 kPa CO_2_; orange triangles: condition b, CO_2_ starvation in the presence of 81 kPa H_2_. Open symbols: O_2_ exposure for 1 h; filled symbols: no O_2_ exposure. Error bars represent the standard deviation of data from at least three biological replicates. The dashed lines are linear fits of exponential phase growth, showing similar revival growth rates for the two conditions without O_2_ exposure.


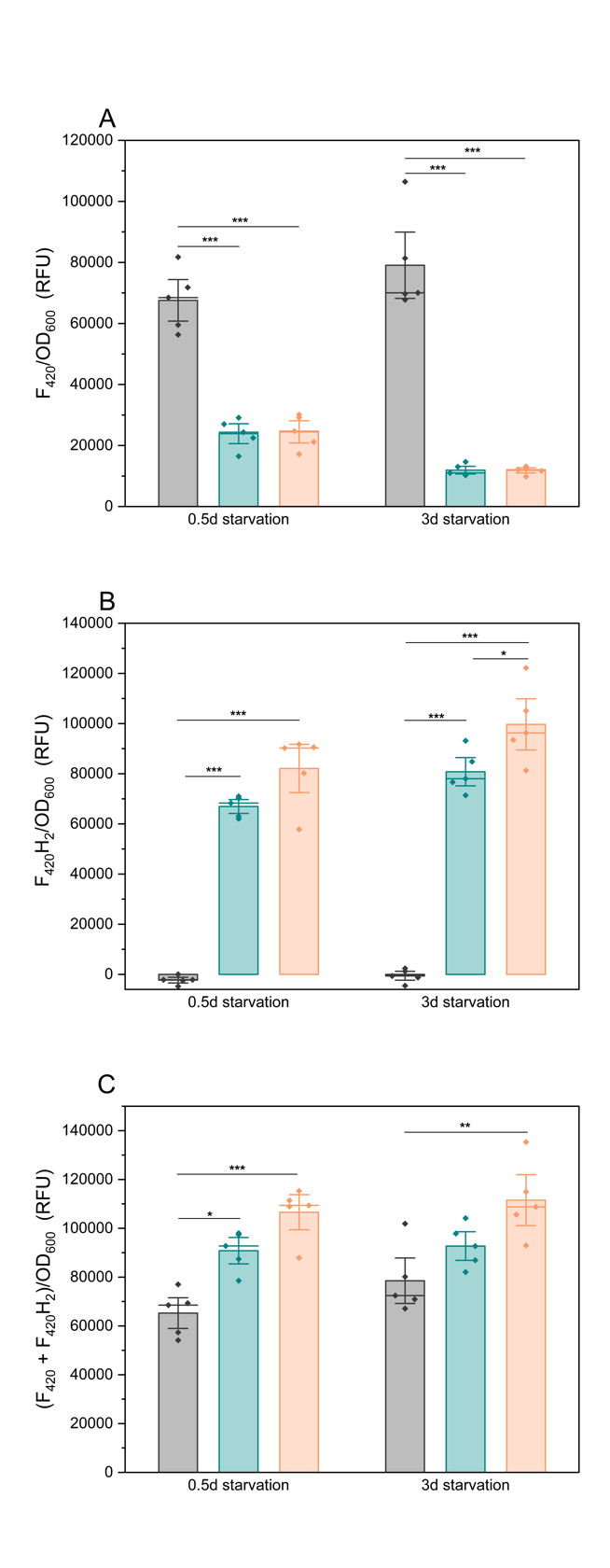


Figure S5. OD_600_-normalized fluorescence intensity of F_420_H_2_ (A), F_420_ (B), and the total F_420_ (C) in *M. maripaludis* at 0.5 and 3 days after the onset of starvation, under 3 gas conditions. Grey: condition a, H_2_ starvation in the presence of 10 kPa CO_2_; green: condition c, CO_2_ starvation in the presence of 20 kPa H_2_; orange: condition b, CO_2_ starvation in the presence of 81 kPa H_2_. Error bars represent the standard deviation of data obtained from at least 5 biological replicates. Tukey’s multiple comparisons test was performed to compare H_2_ and CO_2_ starvations and starvation days for each condition. Stars indicate significant differences. *: P < 0.05, **: P < 0.01, ***: P < 0.001.


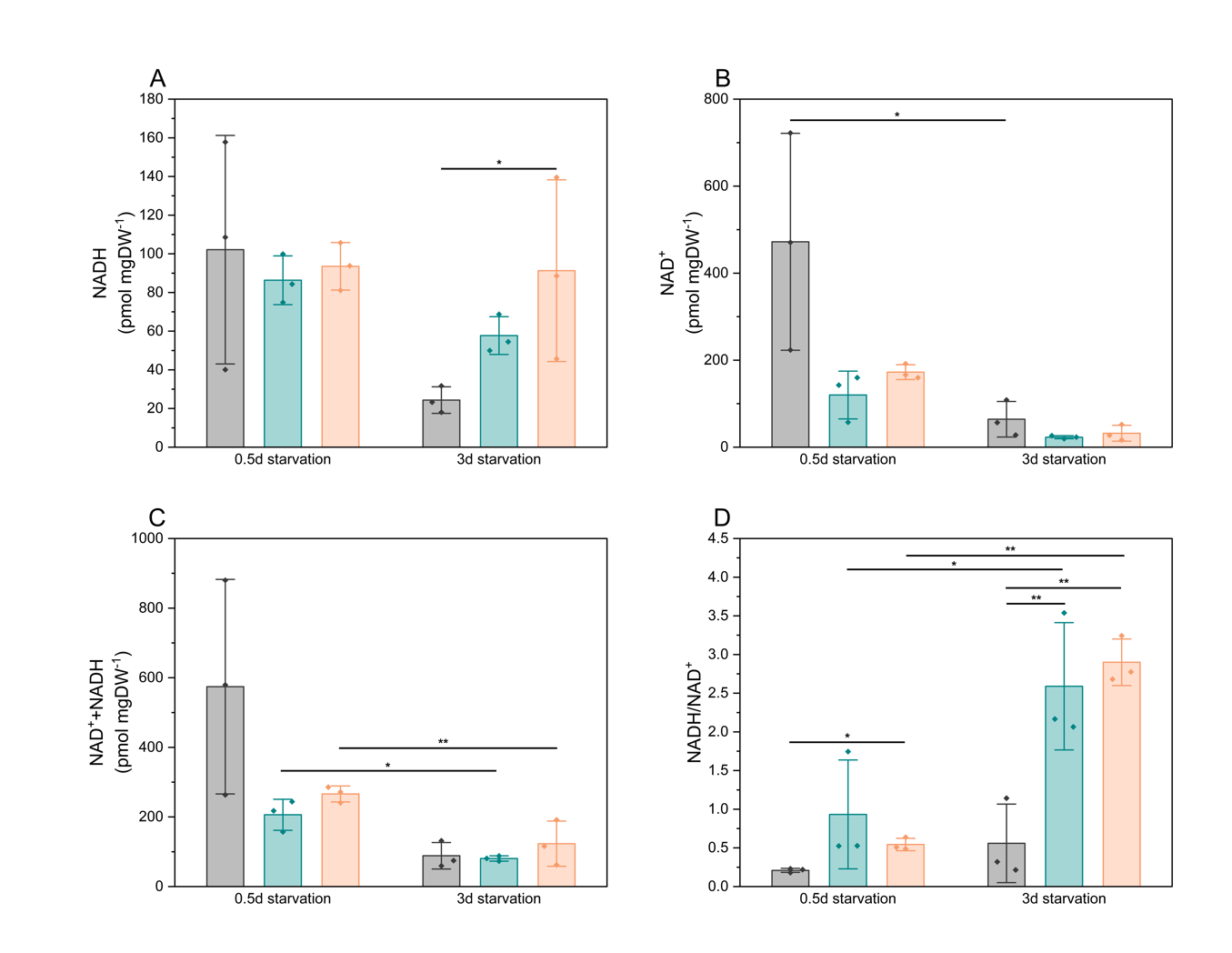


Figure S6. NADH concentration (A), NAD^+^ concentration (B), sum of NADH and NAD^+^ concentration (C), and NADH/NAD^+^ ratio (D) in *M. maripaludis* at the onset of starvation or after 3 days of starvation, under 3 gas conditions. Grey: condition a, H_2_ starvation in the presence of 10 kPa CO_2_; green: condition c, CO_2_ starvation in the presence of 20 kPa H_2_; orange: condition b, CO_2_ starvation in the presence of 81 kPa H_2_. Error bars represent the standard deviation of data from at least three biological replicates. Significance tests were performed to compare H_2_ and CO_2_ starvation, and between starvation days for each condition. Stars indicate significant differences, *: P < 0.05, **: P < 0.01, based on F test of equality of variances followed by post hoc analysis using Tukey’s multiple comparisons test or two-sample t-test (Welch correction).

References

1. Thevasundaram K, Gallagher JJ, Cherng F, Chang MCY. 2022. Engineering nonphotosynthetic carbon fixation for production of bioplastics by methanogenic archaea. Proc Natl Acad Sci USA 119:e2118638119.
